# Morphology of the nervous system of monogonont rotifer *Epiphanes senta* with a focus on sexual dimorphism between feeding females and dwarf males

**DOI:** 10.1186/s12983-019-0334-9

**Published:** 2019-08-07

**Authors:** Ludwik Gąsiorowski, Anlaug Furu, Andreas Hejnol

**Affiliations:** Sars International Centre for Marine Molecular Biology, University of Bergen, Thormøhlens Gate 55, N-5006 Bergen, Norway

**Keywords:** Gnathifera, Neuroanatomy, Sexual dimorphism, CLSM, Meiofauna, Male dwarfism, Protonephridia, Heterochrony

## Abstract

**Background:**

Monogononta is a large clade of rotifers comprised of diverse morphological forms found in a wide range of ecological habitats. Most monogonont species display cyclical parthenogenesis, where generations of asexually reproducing females are interspaced by mixis events when sexual reproduction occurs between mictic females and dwarf, haploid males. The morphology of monogonont feeding females is relatively well described, however data on male anatomy are very limited. Thus far, male musculature of only two species has been described with confocal laser scanning microscopy (CLSM) and it remains unknown how dwarfism influences the neuroanatomy of males on detailed level.

**Results:**

Here, we provide a CLSM-based description of the nervous system of both sexes of *Epiphanes senta*, a freshwater monogonont rotifer. The general nervous system architecture is similar between males and females and shows a similar level of complexity. However, the nervous system in males is more compact and lacks a stomatogastric part.

**Conclusion:**

Comparison of the neuroanatomy between male and normal-sized feeding females provides a better understanding of the nature of male dwarfism in Monogononta. We propose that dwarfism of monogonont non-feeding males is the result of a specific case of heterochrony, called “proportional dwarfism” as they, due to their inability to feed, retain a juvenile body size, but still develop a complex neural architecture comparable to adult females. Reduction of the stomatogastric nervous system in the males correlates with the loss of the entire digestive tract and associated morphological structures.

**Electronic supplementary material:**

The online version of this article (10.1186/s12983-019-0334-9) contains supplementary material, which is available to authorized users.

## Background

Monogononta is a large clade belonging to Rotifera (=Syndermata) with about 1600 species formally described [1]. These microscopic animals inhabit both freshwater and marine environments, and occupy many different ecological niches from being sessile suspension feeders to motile planktonic predators [1, 2]. This ecological diversity is coupled with a vast variety of body plans [3] and morphological adaptations to their particular life style. Despite this variation of monogonont morphology, it is often possible to distinguish three main body regions: 1. head, equipped with a wheel organ or corona, which serves for food capture, sensation, and locomotion, 2. trunk, which contains, among other organs, the characteristic pharynx (mastax) with sclerotized jaws (trophi) and 3. posterior foot with terminal paired toes containing pedal glands used for adhesion to the substrate [1]. Similarly to bdelloids, another large rotiferan clade, monogononts are able to reproduce asexually by producing parthenogenetic eggs. Under non-stressful conditions this type of reproduction dominates [2, 4, 5]. However, unlike bdelloids which are exclusively parthenogenetic [6], most monogonont species also reproduce sexually, often as a response to stressful environmental stimuli [2, 4, 5, 7–10]. Monogonont haploid males are predominantly dwarf and short-living, often with a reduced digestive system, a single testicle, and copulatory organs occupying most of their body [5, 11–13].

The nervous system architecture has been studied in many monogonont species from diverse evolutionary lineages and ecological niches using light microscopy and TEM, as well as histochemical and immunohistochemical techniques combined with epifluorescent and confocal laser scanning microscopy (e.g. [13–34]). Further, gene expression in the developing and juvenile nervous system of monogonont rotifers has recently been studied [35, 36]. However, most of these studies focused on the nervous system of feeding females, whereas the neuroanatomy of dwarf males remains poorly examined. The only available information on the male nervous system dates back to the light microscopy investigation from the beginning of twentieth century [32, 37] and a single histofluorescent labeling of the catecholaminergic structures combined with epifluorescent light microscopy [13]. Neither of these studies provide great resolution of examined structures or detailed comparison of male and female neuroanatomies. So far, the only work based on confocal microscopy that systematically treated sexual dimorphism in monogonont morphology focused on body musculature [12]. Therefore, it remains unknown how male dwarfism influences nervous system architecture in Monogononta.

*Epiphanes* (=*Hydatina*) *senta* (Müller, 1773) was one of the species investigated for general sexual dimorphism by Wesenberg-Lund [37], as well as for sexual dimorphism in musculature by Leasi et al. [12]. It is a relatively large freshwater rotifer, found around the world in littoral habitats of eutrophic water bodies, such as lakes, small ponds, astatic pools and floodplains [10, 38, 39]. Females are relatively stationary, mostly attached or slowly swimming near the substrate, feeding on algae and bacteria, which they filter and collect using the corona [12, 39]. However, they can also ascend to the water column and cases of cannibalism have been observed [12]. The males are about half the length of females [12, 38] and can be found throughout the year (although normally in small densities) in the animal lab cultures (personal observation). Further, the males of *E. senta* display a unique precopulatory mating behavior seemingly sensing and prioritizing eggs of prospective mictic females and then copulate with these females as they emerge from the eggs [10, 39].

In order to test if male dwarfism is coupled with substantial changes in the neuroanatomy, we investigated the nervous system of females and dwarf males of *E. senta* using confocal laser scanning microscopy (CLSM) combined with antibody staining against common nervous system markers (tyrosinated tubulin, acetylated tubulin, serotonin and FMRF-amide). Accordingly, we provide a CLSM-based detailed description of the nervous system in monogonont dwarf males. By comparing it to the nervous system of conspecific females, we can better understand the nature of male dwarfism in Monogononta, as well as infer the impact of this phenomenon on the morphology of one of the most essential organ systems.

## Results

### Taxonomical remark

Schröder and Walsh [38] reported that *E. senta* is a species complex of morphologically almost identical cryptic species, that mostly differ from each other in geographical distribution, details of trophi morphology, and the sculpturing of the resting egg shell. We assume that animals, which we used in our study, represent *E. senta*, however for the sake of future exact taxonomical identification we searched the transcriptome of the investigated species for COX1 sequence. We obtained two sequences of 686 bp each, which differ between each other in 6 nucleotides (either due to intraspecific polymorphism or sequencing inaccuracy) and are made available as Additional file 1.

### General morphology

**Fig. 1:**
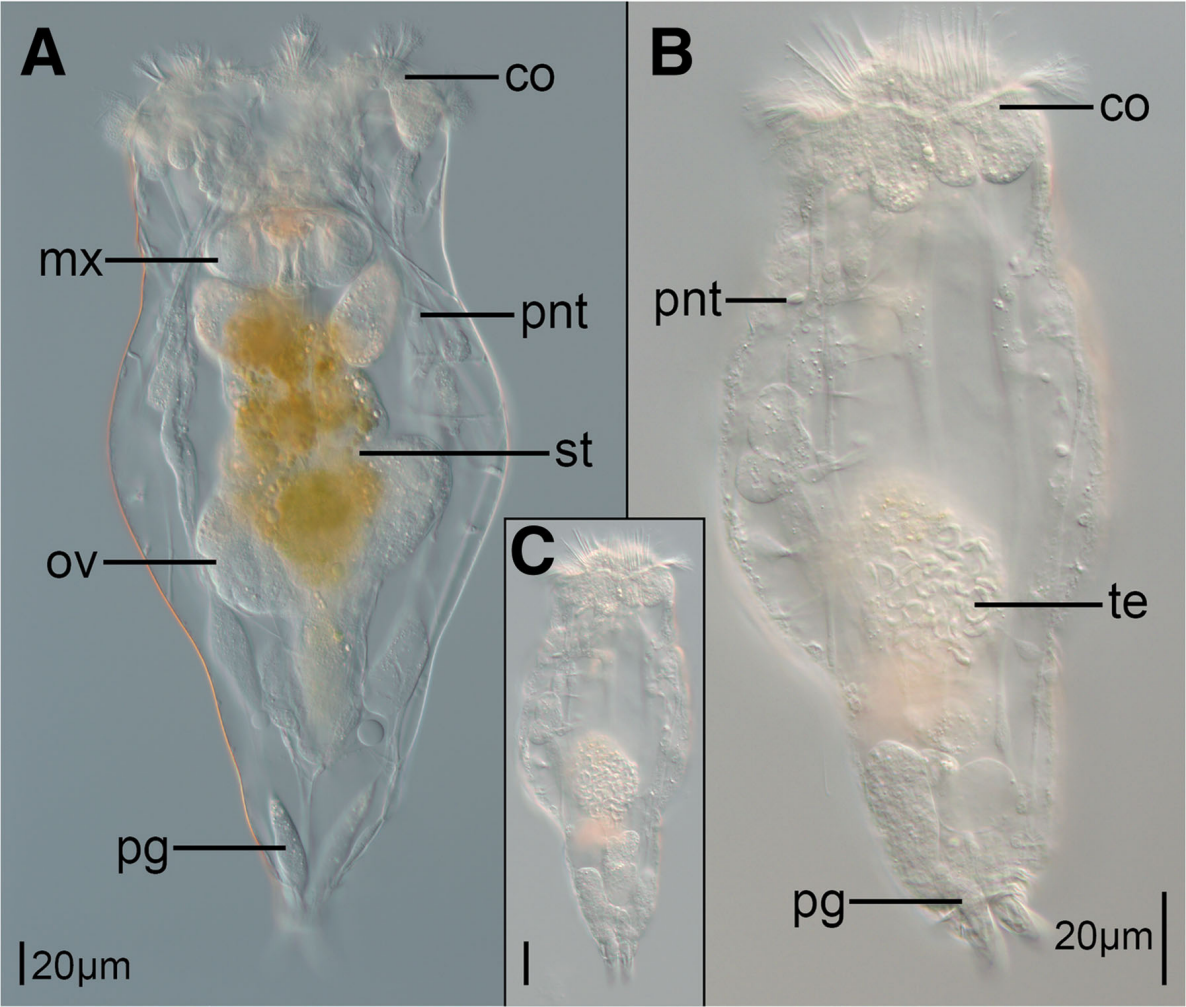
Light micrographs showing sexual dimorphism in *Epiphanes senta*. **a** female, **b** enlarge picture of the male, **c** male in the same scale as female. Abbreviations: *co* corona, *mx* mastax, *ov* ovary, *pg* pedal gland, *pnt* protonephridial terminal organ, *st* stomach, *te* testes with spermatozoa

The body of both sexes of *E. senta* is clearly divided into three regions: head with corona, trunk and foot (Fig. 1). Males and fully developed females clearly differ in body size (Fig. 1a, c), with mean body length of ≈220 μm (*N* = 3) for males and ≈487 μm (*N* = 7) for females. However, newly hatched females are substantially smaller (the smallest measured specimen was 340 μm long) and could be confused with males if solely evaluated by body size. Due to the fact that the body wall of *E. senta* is transparent it is possible to identify most of the internal organs, including gonads, glands and protonephridial terminal organs in both sexes, using light microscopy (LM) (Fig. 1a, b). Even though body shape and proportions are similar between the sexes, males obviously lack any elements of the digestive tract (Fig. 1b). Additionally, a single testicle with individual spermatozoa visible in LM (*te*, Fig. 1b) is found in the posterior part of the male trunk, which makes it easy to distinguish between the sexes regardless of the body size.

### Nervous system of the female

**Fig. 2:**
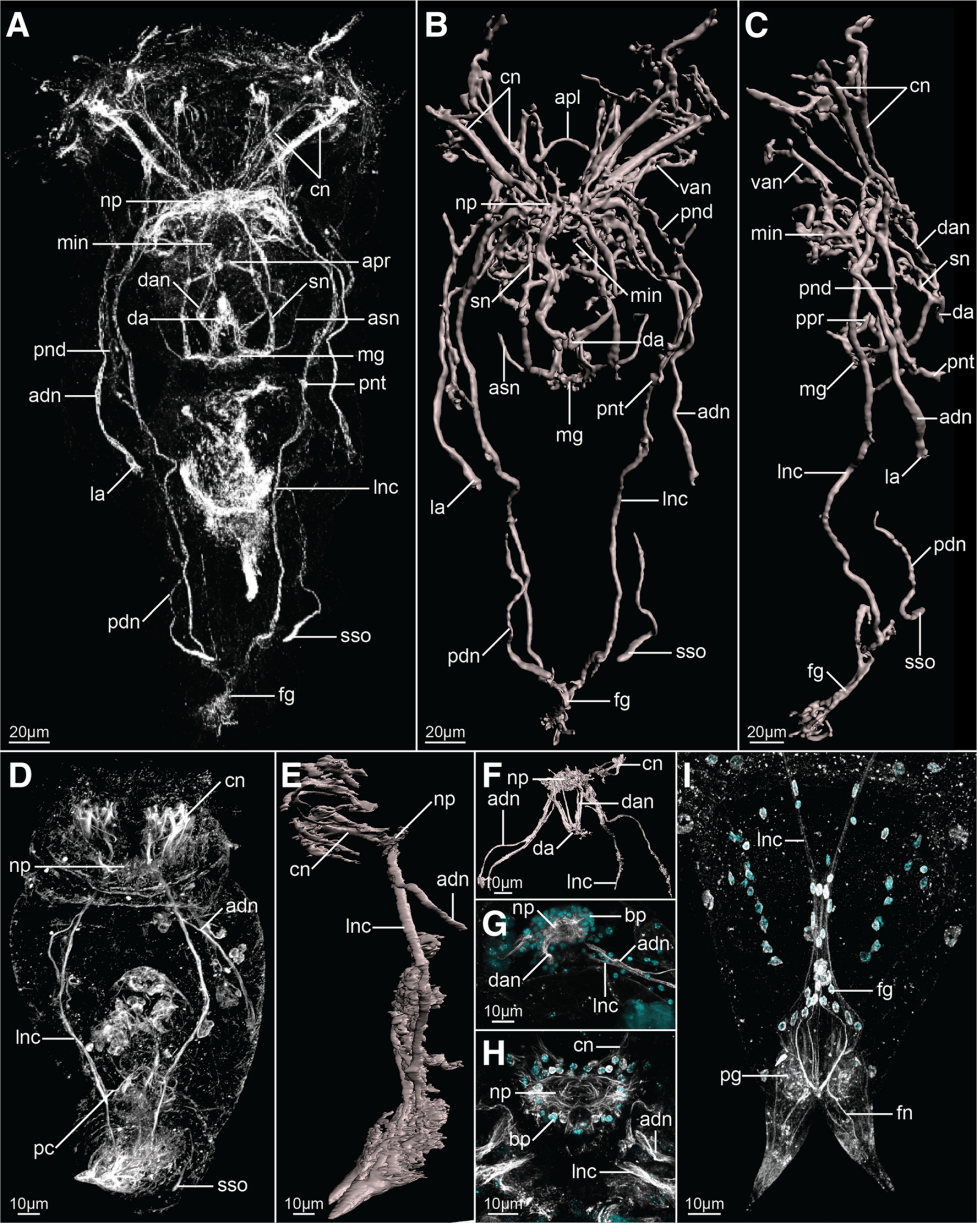
Z-projections (**a**, **d**, **g**–**i**) and 3-D reconstructions (**b**, **c**, **e** and **f**) of the nervous system of *Epiphanes senta* females (**a**–**c**, **h**, **i**) and males (**d**–**g**), visualized with CLSM combined with antibody staining against tyrosinated-tubulin (white) and DAPI staining of cell nuclei (cyan). Entire animals in dorso-ventral (**a**, **b**, **d**) and lateral (**c**, **e**) views. Details of the anterior part of the nervous system (**f**), brain (**g**, **h**) and posterior structures (**i**). In all panels anterior is to the top. Abbreviations: *adn* anterior dorsal nerve, *apl* anterior protonephridial loop, *apr* anterior pharyngeal receptor, *asn* accessory stomatogastric nerve, *bp* brain perikarya, *cn* coronal nerves, *da* dorsal antenna, *dan* nerve of dorsal antenna, *fg* foot ganglion, *fn* foot nerve, *la* lateral antenna, *lnc* longitudinal nerve cord, *mg* mastax ganglion, *min* mouth innervation, *np* neuropile, *pc* posterior commissure, *pdn* posterior dorsal nerve, *pg* pedal gland, *pnd* protonephridial duct, *pnt* protonephridial terminal organ, *ppr* posterior pharyngeal receptor, *sn* stomatogastric nerve, *sso* supraanal sensory organ, *van* ventro-anterior nerve

**Fig. 3:**
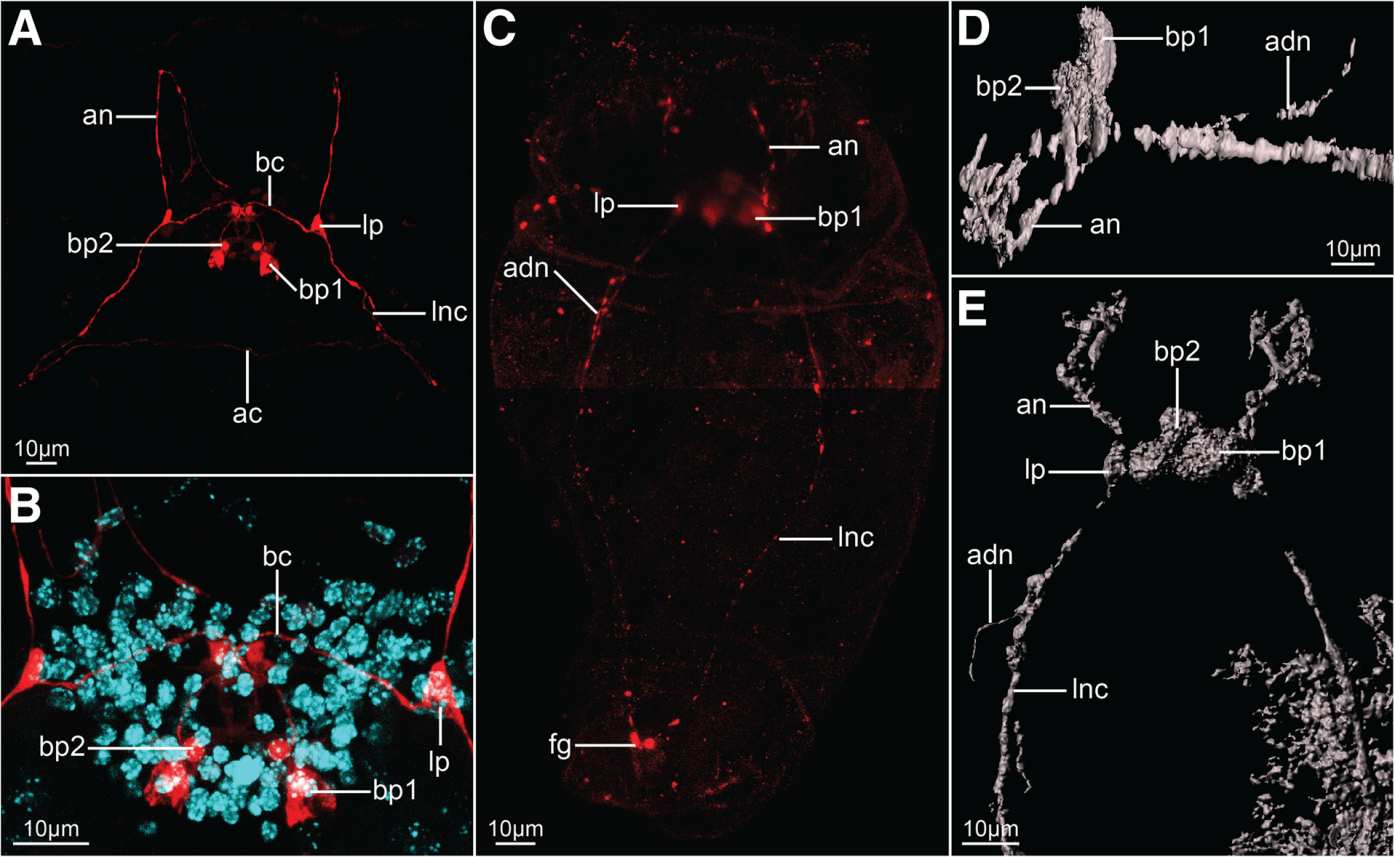
Serotonin-like immunoreactivity in the nervous system of *Epiphanes senta* females (**a**, **b**) and males (**c**–**e**). Z-projections of CLSM (**a**–**c**) showing antibody staining against serotonin (red) and DAPI staining of cell nuclei (cyan) and 3-D reconstructions (**d**, **e**) in dorso-ventral (**a**–**c**, **e**) and lateral (**d**) views. Details of the anterior part of the nervous system (**a**, **d**, **e**) and brain (**b**). Anterior is to the top (**a**–**c**, **e**) and to the left (**d**), dorsal to the top on panel **d**. Abbreviations: *ac* anterior commissure, *adn* anterior dorsal nerve, *an* anterior nerve, *bc* brain commissure, *bp* brain perikarya, *fg* foot ganglion, *lnc* longitudinal nerve cord, *lp* lateral perikaryon

**Fig. 4:**
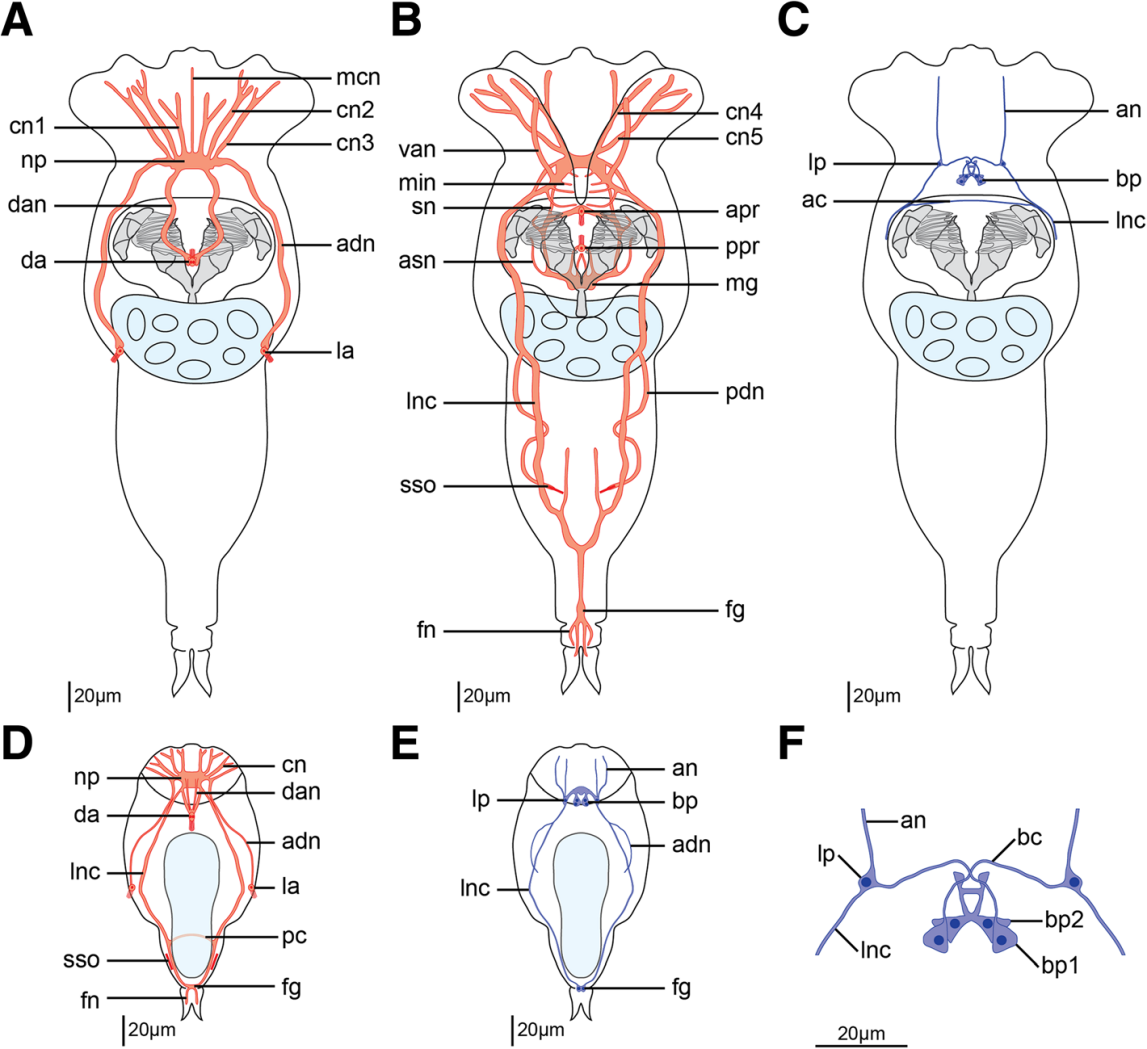
Schematic drawings of the nervous system of *Epiphanes senta* females (**a**–**c**, **f**) and males (**d**, **e**) inferred from tyrosinated tubulin-like immunoreactivity (red) and serotonin-like immunoreactivity (dark blue). Dorsal structures (**a**), ventral structures (**b**), entire body (**c**–**e**) and details of the brain (**f**) in dorso-ventral view with anterior to the top. Abbreviations: *ac* anterior commissure, *an* anterior nerve, *and* anterior dorsal nerve, *apr* anterior pharyngeal receptor, *asn* accessory stomatogastric nerve, *bc* brain commissure, *bp* brain perikarya, *cn* coronal nerves, *dan* nerve of dorsal antenna, *fg* foot ganglion, *fn* foot nerve, *la* lateral antenna, *lnc* longitudinal nerve cord, *lp* lateral perikaryon, *mcn* median coronal nerve, *mg* mastax ganglion, *min* mouth innervation, *np* neuropile, *pc* posterior commissure, *pdn* posterior dorsal nerve, *ppr* posterior pharyngeal receptor, *sn* stomatogastric nerve, *sso* supraanal sensory organ, *van* ventro-anterior nerve

**Fig. 5:**
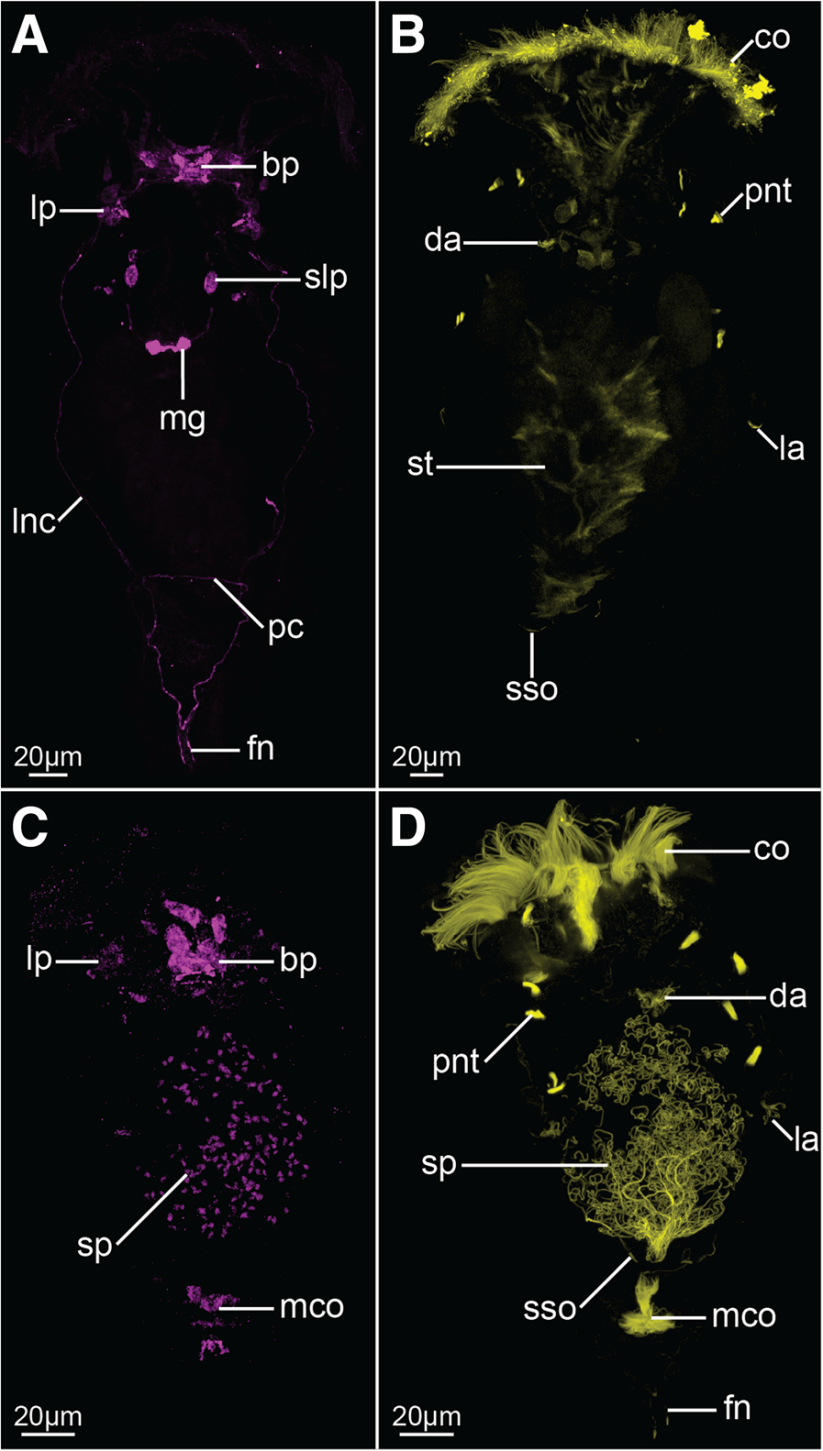
Z-projections showing FMRF-amide-like (**a**, **c**) and acetylated-tubulin-like (**b**, **d**) immunoreactivity in *Epiphanes senta* females (**a**, **b**) and males (**c**, **d**). Dorso-ventral view with anterior to the top on all panels. Abbreviations: *bp* brain perikarya, *co* corona, *da* dorsal antenna, *fn* foot nerve, *la* lateral antenna, *lnc* longitudinal nerve cord, *lp* lateral perikarya, *mco* male copulatory organs, *mg* mastax ganglion, *pc* posterior commissure, *pnt* protonephridial terminal organ, *slp* stomatogastric lateral perikaryon, *sp* spermatozoa, *sso* supraanal sensory organ, *st* stomach

**Fig. 6:**
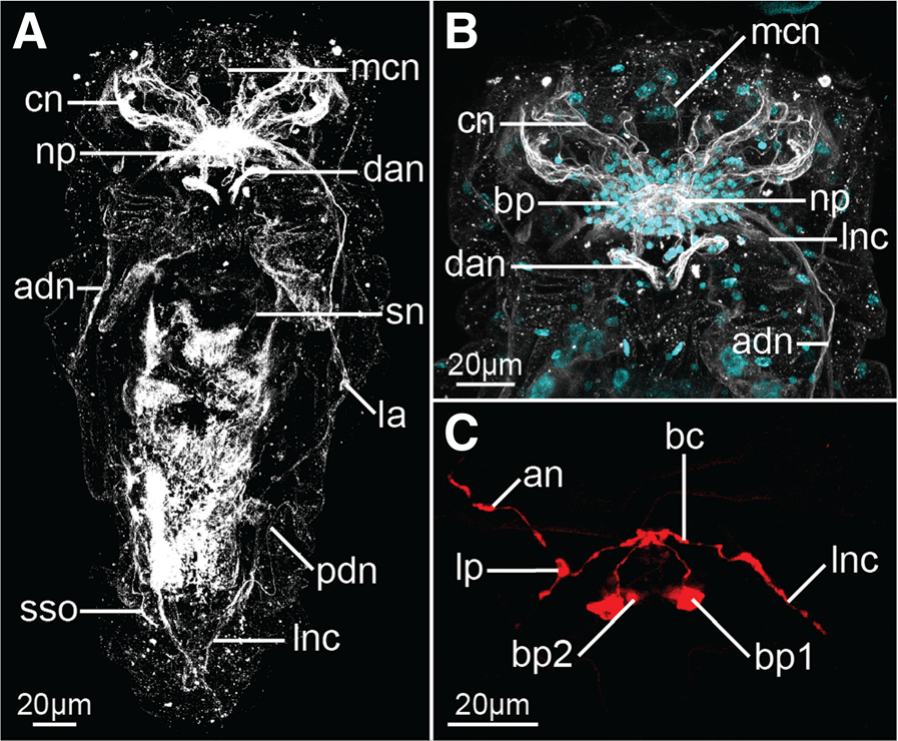
Z-projection showing tyrosinated tubulin-like (**a**, **b**) and serotonin-like (**c**) immunoreactivity, as well as DAPI staining of cell nuclei (cyan, **b**) in juvenile female of *Epiphanes senta*. Dorsoventral view with anterior to the top on all panels. Abbreviations same as on Figs. 2 and 3

The nervous system of feeding females consists of 1) the brain, located in the dorso-posterior part of the head, 2) two longitudinal nerve cords originating laterally in the brain and extending ventro-laterally along the trunk, connected by two trunk commissures and merging posteriorly in the foot ganglion, 3) coronal nerves, 4) peripheral nerves and sensory organs, and 5) stomatogastric nervous system innervating the mastax (Figs. 2a–c, 3a, 4a–c, 5a, b and 6).

Staining against tyrosinated tubulin as well as DAPI staining show that the brain is ellipsoidal (mean length ≈24 μm, mean width ≈58 μm; *N* = 7) and consists of an external layer of perikarya surrounding brain on all sides and internal neuropile (*bp* and *np*, Fig. 2h respectively). Anteriorly, and directly from the brain, 11 coronal nerves (5 paired and one single dorso-median nerve) originate (*cn* and *mcn*, Figs. 2a–c, h, 4 a, b, 6a, b) and innervate large, cushion-shaped cells at the edge of corona (both in trochus and cingulum). Postero-laterally, two thick bundles of neurites emerge from the brain (Fig. 2); one of them (*adn*) is more dorsal and leads to the lateral antennae (*la*). The second bundle gives rise to the thick longitudinal nerve cords (*lnc*) as well as to fine neurites that innervate mouth opening (*min*) and ventro-anterior nerves (*van*) that extend to the ventral part of the corona (Figs. 2a–c, h and 3a, b). Two pairs of nerves originate in the posterior side of the brain: ventrally the stomatogastric nerves (*sn*), and dorsally the nerves of the dorsal antenna (*dan*) (Figs. 2a–c and 3a, b). Staining against serotonin revealed presence of three pairs of serotonin-like immunoreactive (SLIR) perikarya in the brain of female (Figs. 3a, b and 4f), two of which form clusters in the dorso-posterior part of the brain (*bp1* and *2*, Figs. 3a, b and 4f). Neurites of BP1 extends contralaterally, cross each other in the anterior brain, forming the only SLIR commissure of the brain (*bc*) and then connect with the lateral SLIR perikarya (*lp*, Figs. 3a, b and 4f). From each of those bipolar lateral perikarya one SLIR neurite (*an*) extends anteriorly to the corona (note, it is not identical with any of the aforementioned coronal nerves) and a second SLIR neurite (*lnc*) contribute to the lateral nerve cord (Figs. 3a and 4f). FMRF-amide-like immunoreactivity (FLIR) was detected as well in some of the brain perikarya (*bp*, Fig. 5a). However, it was impossible to determine the exact number, identity and connectivity of the FLIR neurons.

Lateral nerve cords (*lnc*, Figs. 2a–c, 3a, 4b, c and 5a) extend along the trunk and posteriorly they merge in the foot ganglion (*fg*), which is a concentration of around 25 perikarya located at the trunk/foot boundary (Fig. 2i). Short nerves extend from the foot ganglion towards pedal glands and tips of the foot toes (*fn*, Figs. 2i, 4b, 5a). At the level of the gonad a single posterior dorsal nerve (*pdn*) originates from each nerve cord and extends dorsally (Figs. 2a–c, 4b). This meandering bundle eventually innervates the supra-anal sensory organ (*sso*) in the dorso-posterior part of the trunk (Figs. 2a–c, 4b and 5b). Some of the neurites of the lateral nerve cords are SLIR (only in the anterior portion of the cord, Figs. 3a, 4c) and FLIR (Fig. 5a). The SLIR neurites form an anterior commissure connecting longitudinal cords ventrally at the level of the anterior mastax, whereas FLIR neurites form a posterior commissure at the level of hindgut (*ac*, Figs. 3a, 4c and *pc*, Fig. 5a, respectively). There are clusters of several FLIR perikarya related with the anterior section of each nerve cord laterally to the mouth opening (*lp*, Fig. 5a), but there are no SLIR perikarya related with the lateral nerve cords.

The stomatogastric nervous system (SNS) makes up a large portion of the female nervous system. The mastax ganglion (*mg*) is a central element of the SNS located in the posterior part of the mastax (Figs. 2a–c, 4b and 5a). Tyrosinated tubulin-like immunoreactivity was detected in the central portion of the ganglion, whereas two of its perikarya are FLIR. A pair of stomatogastric nerves (*sn*) connects mastax ganglion with the ventro-posterior brain and give rise to short accessory stomatogastric nerves (*asn*) innervating lateral portion of the mastax (Figs. 2a–c, 4b). At least one pair of large FLIR perikarya is present along the stomatogastric nerves (*slp*, Fig. 5a). Two pharyngeal unicellular ciliated receptors are associated with the SNS: one (*apr*) in the anterior mastax, with cilia protruding posteriorly, and the second (*ppr*) in the posterior mastax with cilia protruding anteriorly, between jaws (Figs. 2a–c and 4b). The anterior pharyngeal receptor connects to the stomatogastric nerves, whereas the posterior one connects directly with the mastax ganglion. There are no SLIR structures in the SNS of *E. senta* females.

Staining against tyrosinated and acetylated tubulin revealed five sensory organs on the surface of the female body that connect with the nervous system. Three of them (unpaired dorsal antenna in the posterior head and paired lateral antennae in the middle portion of the trunk) are multiciliated epidermal cells (*da* and *la*, Figs. 2a–c, 4a and 5b) with at least one cell nucleus (in each lateral antennae) and two cell nuclei (in dorsal antenna). The paired supra-anal sensory organs present the second type of sensory organs positioned laterally to the anal opening on the dorsal side of the body (*sso*, Figs. 2a–c, 4b and 5b). The individual cilia are never visible in the organ that appears as a solid, seemingly anucleated, elongated structure with a strong immunoreactivity, which seems to be directly continuous with the posterior dorsal nerve.

Moreover, part of the excretory system was stained with antibodies against tyrosinated and acetylated tubulin. Acetylated tubulin-like immunoreactivity was detected in four pairs of ciliated terminal organs of the protonephridial system (*pnt*, Fig. 5b), showing a typical monogonont organization with all particular cilia of each organ forming a common flame. Terminal organs were also stained (albeit weakly and not in all specimens) with antibodies against tyrosinated tubulin (*pnt*, Fig. 2a–c); additionally, tyrosinated tubulin-like immunoreactivity was detected in the protonephridial ducts of some specimens (*pnd*, Fig. 2a–c) revealing that the ducts are anteriorly connected by the loop positioned anteriorly to the brain (*apl*, Fig. 2b).

We additionally investigated one freshly hatched juvenile female with antibodies against tyrosinated tubulin and serotonin as well as nuclear DAPI staining (Fig. 6). All of the aforementioned tyrosinated TLIR and SLIR structures have been confirmed and the nervous system show the same arrangement and level of complexity as in fully grown females, while the number of cell nuclei (also in the brain) does not seem to be reduced compared to older specimens (compare Figs. 6a, b, c with 2a–c, h and with 3a, b).

### Nervous system of the male

Similarly to the females, the nervous system of male *E. senta* consists of a frontal brain, longitudinal nerve cords, coronal and peripheral nerves and sensory structures (Figs. 2d, e, 3c–e, 4d, e and 5c, d). The stomatogastric nervous system is, however, entirely lacking in the males.

The male brain has a similar shape and length to the female’s brain (mean length ≈20 μm, mean width ≈37 μm; *N* = 2), although it is narrower and seems to be more compact (compare Fig. 2 g with h). As in females it is clearly divided into an outer layer of perikarya and an internal neuropil (*bp* and *np*, Fig. 2g, respectively). Anteriorly, coronal nerves protrude from the brain (*cn*, Figs. 2 d–f, 4d), yet their exact number was difficult to determine due to the aforementioned compactness. Similar as in the females, two pairs of thick nerves emerge laterally from the brain: one of them continues as a pair of anterior dorsal nerves (*adn*) and connects to the lateral antennae, whereas the other continues posteriorly as the longitudinal nerve cords (*lnc*) (Figs. 2 d–f, 4d). Dorso-posteriorly two nerves (*dan*) connect the neuropile with the dorsal antenna (Figs. 2f, 4d). There are three pairs of SLIR perikarya, which occupy similar positions as those found in the female brain (Figs. 3c–e, 4e), however, they are so densely packed that it is impossible to resolve their exact connection with each other. Nevertheless, the anterior SLIR neurites (*an*) and SLIR neurites of the longitudinal nerve cords (*lnc*) connect laterally to this cluster of SLIR brain perikarya (Figs. 3c–e, 4e). FMRF-amide-like immunoreactivity was also detected in some of the brain cells (*bp*, Fig. 5c).

Longitudinal nerve cords (*lnc*) extend from the brain to the foot ganglion (Figs. 2d, e, 3c and 4 d, e), but unlike in females they are SLIR along their entire length (Figs. 3c, 4e) but not FLIR (Fig. 5c). Short foot nerves protrude from the foot ganglion toward the tips of the toes (*fn*, Figs. 4d, 5d). The lateral clusters of weakly FLIR perykarya are present at the anterior portion of the cords (*lp*, Fig. 5c), whereas pair of SLIR perikarya can be detected in the foot ganglion (fg, Figs. 3c, 4e). We did not manage to detect the anterior commissure with any of our immunostainings, but the posterior one exhibits tyrosinated tubulin-like immunoreactivity (*pc*, Figs. 2d, 4d), but no FMRF-like immunoreactivity as in females (Fig. 5c). A pair of fine SLIR neurites extends dorsally from the lateral cords and continues along the anterior dorsal nerves, which lead to the lateral antennae (*adn*, Figs. 3c–e, 4e); however, they do not reach the sensory organs themselves. The posterior dorsal nerves were not directly detected, but the supra-anal sensory organs were visible with staining against acetylated and tyrosinated tubulin (*sso*, Figs. 2d, 5d). The weakly tyrosinated tubulin-like immunoreactive (TLIR) nerve innervates each of the supra-anal organs and, even though its connection to the lateral cords was not possible to trace, we assume that it represents the male counterpart of the female posterior dorsal nerve.

Five sensory organs were detected on the external surface of the male *E. senta* as they are in the female: an unpaired dorsal antenna on the posterior part of the head, the lateral antenna in the middle of the trunk, and the supra-anal sensory organs in the posterior part of the trunk (*da*, *la* and *sso*, Figs. 2d, f, 4d and 5d, respectively). All sensory organs seem to have a similar arrangement and innervation as their counterparts in the female. Four pairs of terminal organs of the male protonephridial system show strong acetylated tubulin-like immunoreactivity and a typical flame-like organization of cilia (*pnt*, Fig. 5d). Additionally, a strong acetylated tubulin-like immunoreactivity was detected in the corona, male copulatory organs and in the sperm flagella (*co*, *mco* and *sp*, Fig. 5d, respectively).

## Discussion

### Differences between females and males of *E. senta*

The configuration of the nervous systems of both sexes of *E. senta* is highly similar on both a general and detailed level. The most pronounced difference is related to the complete reduction of the stomatogastric nervous system (SNS) in males. Additionally, the anterior commissure connecting the lateral nerve cords on ventral side was not detected in males. Apart from those two structures lacking in males, we found counterparts of all female nervous structures in the dwarf males. Our observation of the similarity in the nervous system of females and dwarf males are in agreement with LM observation by Wesenberg-Lund, who described rectangular brain, coronal nerves, dorsal and lateral antennae and lateral nerve cords emerging from the brain in dwarf males of *Epiphanes* (*=Hydatina*) *senta* [37]. Previous investigation of the sexual dimorphism in musculature of *E. senta* and *Brachionus manjavacas* [12] showed that males and females have almost identical somatic musculature and differ mostly in the lack of the mastax musculature in males. This muscular similarity is possibly reflected in the comparable neural innervations here found.

The female brain of *E. senta* is approximately two times larger than the male brain by measuring the area of the ellipse appointed by the widest and longest axes of the brain as an indicator of the brain size (area of the widest section of the female brain ≈1093μm^2^, area of the same section in male ≈581μm^2^, ratio: 1.88). This roughly corresponds with the body size difference between females and males (ratio of the mean body length between sexes: 2.21). However, the cell nuclei in the male brain seem to be more densely packed than the nuclei in the female brain, and though not counted herein the exact number of cell nuclei might be actually similar.

Although the general architecture of the nervous system is similar between the two sexes, there are interesting differences in the immunoreactivity of particular structures (Table 1). For instance the lateral nerve cords exhibit FMRF-amide-like immunoreactivity in females but not in males. While on the other hand, their posterior fragments (including the posterior foot ganglion) show serotonin-like immunoreactivity in males but not in females. Further differences in the immunoreactivity are evident for the innervation of lateral antennae, posterior commissure of lateral nerve cords and foot nerves (Table 1). Those differences might indicate that despite a similar morphology, particular elements of the male and female nervous system might vary in their neurophysiology and possibly also in function.

**Table 1:** Summary of the differences between females and males in the immunoreactivity detected in particular morphological structures

Structure	Sex	Immunoreactivity			
		Tyrosinated tubulin-like	Acetylated tubulin-like	Serotonin-like	FMRF-amide-like
Longitudinal nerve cords	Female	+	–	anterior	+
	Male	+	–	entire	–
Posterior commissure	Female	–	–	–	+
	Male	+	–	–	–
Anterior dorsal nerve	Female	+	–	–	–
	Male	+	–	+	–
Foot ganglion	Female	+	–	–	–
	Male	+	–	+	–
Foot nerves	Female	+	–	–	+
	Male	+	+	–	–

In addition to the nervous system, we also visualized portions of the excretory organs, including terminal organs (in both sexes) and protonephridial ducts (in females). Both sexes have four pairs of terminal organs with vibratile ciliary flames, which contrasts with two pairs described by Martini based on his LM observation [28], but conforms to descriptions provided by Wesenberg-Lund [37]. The terminal organs have typical monogonont organization with several cilia forming a common unison flame (e.g. [40]). The movement of those flames was observed in both sexes in LM examinations of living specimens. In females we found an anterior loop connecting nephridial ducts anteriorly to the brain, the structure known from the literature as Huxley’s anastome [1], which has also been described in *E. senta* females and males [28, 37]. The observed details of protonephridia indicate that next to the musculature and nervous system the excretory organs of both sexes are functional and share a similar architecture.

### Male dwarfism in Monogononta

Male dwarfism is a relatively widespread phenomenon present in many organisms [41]. Among Spiralia (to which rotifers belong [42–45]) it has been reported in e.g. Cycliophora [46–50], Orthonectida [51], which are now considered parasitic annelids [52–54], some octopods [55] and in several annelid clades including some dinophilids [56–58], *Osedax* [59–61], Spionidae [62, 63] and bonellid echiurans [64]. Presence of dwarf males has also been proposed as an explanation for the occurence of resting eggs and seeming lack of males in Subantarctic and Arctic populations of *Limnognathia maerski*, the sole representant of Micrognathozoa, a sister taxon of Rotifera [65]. There are two proposed mechanisms responsible for the origin of dwarfism: heterochrony (in the form of progenesis or proportioned dwarfism) and gradual miniaturization [66–72]. Those two evolutionary processes are reflected in the morphology of the dwarfed forms, including their nervous system and musculature [56]. The progenetic animals resemble earlier (larval or juvenile) developmental stages of normal-sized counterparts, whereas proportionally dwarfed animals would be decreased in size but otherwise resemble their normal-sized counterparts in shape and development. Lastly, dwarfs as a result of gradual miniaturization lack many characters typical for non-reduced specimens and often they have numerous anatomical adaptations to the reduction, which do not bear any obvious homology to neither larval nor adult structures of the normal-sized specimen [56, 67]. The morphology of some spiralian male dwarves, such as bonellid echiurans and *Osedax* [60, 61, 64], resemble larvae, which indicates progenetic evolution, whereas dwarf males of cycliophorans, orthonectids, *Dinophilus gyrociliatus* (Dinophilidae) and *Scolelepis laonicola* (Spionidae) are not similar to the early developmental stages of their female counterparts and rather originated through a series of evolutionary losses or a more complex mix of heterochronous and non-heterochronous evolutionary events [48, 51, 56, 63].

At the moment of hatching, the male of *E. senta* is of similar size and complexity as female and only its digestive system with associated structures (mastax musculature, stomatogastric nervous system) is reduced ([12, 37], this study). The post-hatching growth of rotifer females is achieved mostly through increase in the size of the cells but not their number [1, 73], thus the feeding females become larger while their neuroanatomy, musculature, excretory system and general shape remain comparable to that of the juvenile females or dwarf, non-feeding males [37]. Hence, with the exception of the digestive tract, the dwarfism of the male results from decrease and eventual arrest of the growth rate, caused by the lack of digestive system. This phenomenon can be categorized as proportional dwarfism [66, 69–72], with monogonont males having changed their size but not their shape relative to the normal-sized females, due to the decreased growth rate. On the other hand, in case of Monogononta the only differences between proportional dwarfism and progenesis (sensu Gould 1977 and Alberch et al. 1979 [66, 69–71]) would be onset of sexual maturity. The reason is that “shape hand” of the Gould’s clock model of heterochrony remains still in case of postembryonic development of monogonont rotifers, as general shape and cellular complexity of post-hatching monogonont female remain unchanged. If males are sexually matured at the moment of hatching, while females remain sexually immature until they reach a certain size, then the observed phenomenon would rather conform to the definition of progenesis. If both sexes reach sexual maturity at approximately similar developmental time points, then proportional dwarfism, as proposed here, would persist as the best explanation of the observed size differences. Further investigation of the development of reproductive system, optimally combined with cell lineages studies, would be needed to ultimately ascertain.

Both feeding and non-feeding males have been reported from monogononts and apparently the species’ ecology, and not phylogeny, seems to predominantly explain presence of one or the other form [11]. This indicates that the loss of the digestive system in the male (and subsequent dwarfism) might be reversible in Monogononta. Evolutionary reversal from dwarf progenetic males to normal-sized organisms was already reported in the bone eating annelid *Osedax priapus*, proving that transition to male dwarfism is evolutionarily labile and not necessarily unidirectional [59].

### Nervous system of *E. senta* females – a comparative view

Martini [28] described the morphology of *Epiphanes* (*=Hydatina*) *senta* females using LM on intact specimens and histological sections. His description includes, among others, a detailed reconstruction of the nervous system. Results from our investigation show close resemblance with those of Martini [28]. Similarly, Leasi et al. [12] found their CLSM-based reconstruction of musculature congruent with the LM-based reconstruction of Martini. The only neural structure, which Martini did not describe and we revealed in our study, is the thin anterior commissure connecting the longitudinal nerve cords ventrally to the mastax.

The general neuroarchitecture in monogonont females is quite conserved [1, 21, 27] and our reconstruction of the neuroanatomy of *E. senta* females conforms to the generalized plan of the rotifer nervous system. All of the structures, which we hereby described for the female, have been reported in some rotifer species in the previous investigations. There are, however, some aspects of the rotifer nervous system that need an additional discussion.

So far, serotonin and FMRF-amide have been used the most extensively as nervous system markers in rotifers, and comparison of immunoreactivity patterns of those two markers is possible for a broad range of taxa [20–23, 27, 74]. Similarly, as in other Monogononta [20, 21, 27], FMRF-amide-like immunoreactivity seems to be more widely distributed than serotonin-like immunoreactivity in the nervous system of *E. senta* females. However, at the same time the exact connectivity of FLIR perikarya is impossible to trace, whereas connectivity of SLIR perikarya can be reconstructed [21]. Therefore, those two markers should be used for different purposes – the first one allows general but imprecise staining of the large portion of the nervous system, whereas the other allows reconstruction of only the small fraction of the system, but with very accurate cellular resolution.

The serotonin-like immunoreactivity in SNS has been reported for all Ploima species investigated thus far [21, 27], but is apparently absent in all examined Gnesiotrocha [20, 22, 23], a discrepancy that has been stressed as an important difference between those two clades of Monogononta [20]. However, we did not detect serotonin-like immunoreactivity in the SNS of *E. senta* (which is phylogenetically nested within Ploima), which indicates that serotonin-like immunoreactivity in SNS is a homoplastic character in monogonont rotifers similar to what has been reported for the relatively closely related Gnathostomulida [75].

In the available literature there is also some disagreement regarding connections between SNS and the central nervous system in Rotifera. In the older literature, the stomatogastric nerves have been described as directly connecting to the brain (e.g. [28, 76]), an arrangement which has been confirmed by Hochberg [21] in his CLSM study on *Notommata copeus*. On the other hand, the alternative connection to the lateral nerve cords has been also reported in *N. copeus* and *Asplanchna herricki* [21, 27]. In our investigation we found a thick stomatogastric nerve connecting the mastax ganglion directly to the ventro-posterior brain of the female of *E. senta* and no evidence of the connection between SNS and longitudinal cords. The pharynx-related ganglion (or at least condensation of neuronal perikarya [77]) connecting directly to the brain has also been reported in other Gnathifera, i.e. Gnathostomulida [75] and Micrognathozoa (where the exact connection of the ganglion to the brain has not been clearly demonstrated [78]) as well as in Chaetognatha [79]. According to the recent phylogenies Gnathifera and Chaethognatha seem to form a clade [43, 45], and presence of the pharyngeal ganglion directly connecting to the brain has been already proposed as autapomorphy of Chaetognatha+Gnathifera [75].

## Conclusions

We provide a CLSM-based description of the sex-related differences in the nervous system of the monogonont rotifer, exemplified by the well-studied *Epiphanes senta*. The neuroanatomy of both sexes is congruent and shows similar levels of complexity, though the male nervous system is more compact and lacks the stomatogastric part due to the reduction of the digestive tract. Additionally, some of the nervous structures display different immunoreactivities between the sexes, possibly indicating divergence in neurophysiology and function. Comparison of nervous system, musculature and excretory organs between feeding females and dwarf males suggest that male dwarfism in Monogononta reflects the heterochronous phenomenon of proportional dwarfism caused by decreased size growth rate (but not proportional decrease in shape growth rate), which again is related to the reduction of the digestive system.

## Methods

### Animals culturing and fixation

The animals were ordered from a commercial provider of aquatic microinvertebrates (www.sciento.co.uk) in September 2015 and cultured in Jaworski’s medium at 20 °C and a 14:10 h light:dark cycle. The medium was refreshed every two weeks and the animals were fed ad libitum with the algae *Rhodomonas* sp., *Cryptomonas* sp., and *Chlamydomonas reinhardtii*. Under those conditions both females and males are present in the cultures so there is no need for induction of mixis.

The individual animals were transferred with pipette from cultures to an embryo dish with Jaworski medium; feeding females were starved over night. Prior to fixation, the animals were relaxed for approximately 10 min with a solution of 1% bupivacaine and 10% ethanol in culturing medium. Thereafter they were fixed for 1 h in 4% paraformaldehyde solution at room temperature and subsequently rinsed several times with phosphate buffered saline (PBS) with 0.1% Tween-20.

### Immunohistochemistry

After several washes in PBT (PBS + 0.1% Tween-20 + 0.1% bovine serum albumin) animals were preincubated for 30 min at room temperature in PTx + NGS (5% Normal Goat Serum in PBS + 0.1% Triton X-100) and then incubated overnight at 4 °C in primary antibodies (mouse anti acetylated tubulin, Sigma T6793 or mouse anti tyrosinated tubulin, Sigma T9028 and rabbit anti serotonin, Sigma S5545 or rabbit anti FMRF-amide, Immunostar 20091) dissolved in PTx + NGS in 1:500 concentrations. The animals were then rinsed several times in PBT, preincubated for 30 min at room temperature in PTX + NGS and incubated overnight at 4 °C in secondary antibodies (goat anti-mouse conjugated with AlexaFluor647 and goat anti-rabbit conjugated with AlexaFluor488, Life Technologies) dissolved in Ptx + NGS in 1:250 concentrations. Eventually, the animals were rinsed several times in PBT, stained for cell nuclei with DAPI (1:1000 solution in PBS for 40 min) and mounted in 80% glycerol.

Altogether 19 specimens were investigated – six males (three with antibodies against tyrosinated tubulin and serotonin and three with antibodies against acetylated tubulin and FMRF-amide) and 13 females (seven with antibodies against tyrosinated tubulin and serotonin and six with antibodies against acetylated tubulin and FMRF-amide).

### Microscopy and image processing

Mounted specimens were scanned in Leica SP5 confocal laser scanning microscope. Z- stacks of scans were projected into 2D images and 3D reconstructions in IMARIS 9.1.2, which was also used to conduct all the measurements. Schematic drawings based on Z- stacks of scans were made in Adobe Illustrator CS6. Additionally, some living animals anesthetized with bupivacaine solution were photographed with Zeiss Axiocam HRc connected to a Zeiss Axioscope Ax10 using bright-field Nomarski optics. CLSM and light microscopy images were adjusted in Adobe Photoshop CC 2015 and assembled in Adobe Illustrator CS6.

## Additional file

Additional file 1: Two sequences of the COX1 gene obtained from the transcriptome of the investigated rotifer. (TXT 3 kb)

## Data Availability

Confocal Z-stacks used for the descriptions and 3-D reconstructions provided in this study are freely available in MorphDBase [80] via hyperlinks: • female *E. senta*, tyrosinated TLIR structures: www.morphdbase.de/?L_Gasiorowski_20190604-M-17.1) • male *E. senta*, tyrosinated TLIR structures: (www.morphdbase.de/?L_Gasiorowski_20190604-M-14.1) • female *E. senta*, SLIR structures: (www.morphdbase.de/?L_Gasiorowski_20190604-M-18.1) • male *E. senta*, SLIR structures: (www.morphdbase.de/?L_Gasiorowski_20190604-M-22.)1 • female *E. senta*, FLIR structures: (www.morphdbase.de/?L_Gasiorowski_20190604-M-15.1) • male *E. senta*, FLIR structures: (www.morphdbase.de/?L_Gasiorowski_20190604-M-20.1) • female *E. senta*, acetylated TLIR structures: (www.morphdbase.de/?L_Gasiorowski_20190604-M-13.1) • male *E. senta*, acteulated TLIR structures: (www.morphdbase.de/?L_Gasiorowski_20190604-M-19.1) • female *E. senta*, cell nuclei visualized with DAPI: (www.morphdbase.de/?L_Gasiorowski_20190604-M-16.1) • male *E. senta*, cell nuclei visualized with DAPI: (www.morphdbase.de/?L_Gasiorowski_20190604-M-21.1)
